# An Odontogenic Cutaneous Sinus Tract Mimicking a Mandibular Neoplasm: Diagnostic Challenges and Radiologic Correlation

**DOI:** 10.7759/cureus.110677

**Published:** 2026-06-11

**Authors:** Marij W Janjua, Immad Shah, Thomas G Wiedemann, Adnan Shah

**Affiliations:** 1 Oral and Maxillofacial Surgery, New York University, New York City, USA; 2 Oral and Maxillofacial Surgery, Health Sciences Center Winnipeg, University of Manitoba, Winnipeg, CAN

**Keywords:** cutaneous malignancy, mandibular molar, mandibular neoplasm, maxillofacial surgery, nodular lesion, odontogenic fistula, sinus tract

## Abstract

Odontogenic cutaneous sinus tracts (OCSTs) are an uncommon consequence of chronic dental infection in which inflammatory drainage extends from a periapical source through bone and soft tissues to the skin surface. Although odontogenic infections typically present with intraoral symptoms such as pain, swelling, or vestibular drainage, chronic lesions may occasionally manifest as extraoral nodules, cysts, or draining sinuses. Because many patients experience little or no dental discomfort, these lesions are frequently misdiagnosed as dermatologic, infectious, or neoplastic conditions.

The reported incidence of OCSTs is low, and diagnosis is often delayed because patients initially seek care from primary care physicians, dermatologists, otolaryngologists, or surgeons rather than dental providers. Common misdiagnoses include epidermoid cysts, furuncles, pyogenic granulomas, salivary gland pathology, chronic osteomyelitis, and cutaneous malignancies. Failure to identify the underlying dental etiology may result in repeated antibiotic therapy, biopsies, surgical excisions, and prolonged patient morbidity.

Advanced imaging modalities, including cone beam computed tomography (CBCT) and contrast-enhanced computed tomography (CT), can provide valuable diagnostic information when conventional examination is inconclusive or when concern exists for more serious pathology. We present the case of a chronic painless mandibular mass initially investigated for possible malignancy that was ultimately diagnosed as an OCST arising from a mandibular molar with cortical perforation and extraoral extension.

## Introduction

Odontogenic cutaneous sinus tracts (OCSTs) represent an uncommon extraoral manifestation of chronic dental infection in which inflammatory drainage extends from a periapical source through cortical bone and soft tissue to the skin surface. Although odontogenic infections most commonly drain intraorally, persistent infections may occasionally follow the path of least resistance and present as extraoral nodules, cystic lesions, draining sinuses, or chronic facial masses. Because many patients experience minimal dental symptoms, these lesions are frequently misdiagnosed and may undergo prolonged medical, dermatologic, or surgical evaluation before the underlying dental etiology is recognized. Guevara-Gutiérrez et al. reported similar diagnostic challenges in their review of 75 odontogenic cutaneous fistulas, emphasizing that the atypical extraoral presentation of these lesions frequently contributes to delayed recognition and inappropriate initial management [1].

The clinical presentation of OCSTs is highly variable. Lesions may resemble furuncles, epidermoid cysts, pyogenic granulomas, salivary gland pathology, lymphadenopathy, chronic osteomyelitis, or even cutaneous malignancy. The resulting diagnostic delay often exposes patients to unnecessary antibiotic therapy, biopsies, dermatologic procedures, and extensive medical investigations. Lee et al. similarly reported that odontogenic cutaneous fistulas frequently present with nonspecific cutaneous findings and are commonly misdiagnosed because of their variable clinical appearance and lack of overt dental symptoms [2]. Previous reports have emphasized the importance of maintaining odontogenic infection within the differential diagnosis of persistent facial lesions, particularly when located along the mandibular border or submental region.

Because OCSTs often present without prominent dental symptoms, diagnosis may be delayed and patients frequently undergo evaluation by multiple healthcare providers before the dental origin is recognized. Careful clinical examination combined with appropriate dental imaging is essential for identifying the source of infection and avoiding unnecessary investigations or interventions [3]. We present a case of a chronic painless mandibular cutaneous lesion that was initially investigated for possible malignancy before clinical and radiographic findings established an odontogenic etiology arising from a mandibular molar.

## Case presentation

A 54-year-old female patient was referred for evaluation of a persistent painless lesion involving the right inferior border of the mandible. The patient reported a gradual development of the nodular cutaneous mass over several months. The lesion demonstrated intermittent inflammatory changes but was largely asymptomatic. She denied significant facial swelling, trismus, dysphagia, fever, weight loss, or constitutional symptoms.

Clinical examination revealed a well-circumscribed exophytic nodular lesion involving the right mandibular body region near the inferior border of the mandible. The lesion appeared erythematous and dome-shaped with a smooth surface and measured approximately 7 mm in diameter (Figure 1). No active purulent drainage was observed during examination. Palpation revealed a firm subcutaneous component with mild fixation to underlying tissues. Regional lymphadenopathy was absent.

**Figure 1:**
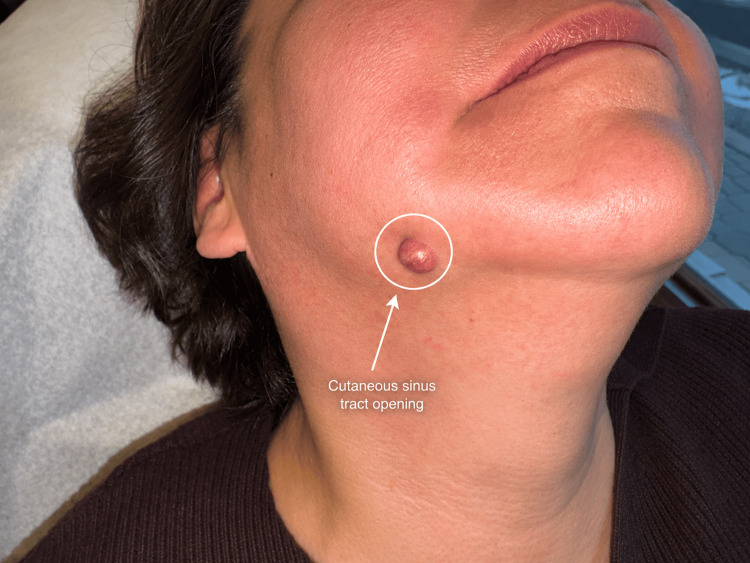
Clinical presentation of the odontogenic cutaneous sinus tract Clinical photograph demonstrating a chronic cutaneous sinus tract presenting as an erythematous exophytic nodular lesion in the right submandibular region. The lesion was associated with a chronic odontogenic infection arising from a mandibular molar and represented the extraoral drainage pathway of the underlying periapical pathology. The sinus tract opening is highlighted by the white circle and arrow.

Intraoral examination demonstrated heavily restored posterior dentition with chronic restorative breakdown involving the right mandibular molar region (Figure 2). No significant vestibular swelling was noted. Mucosal tissues appeared otherwise unremarkable. The patient reported no significant odontogenic pain despite the chronicity of the lesion.

**Figure 2:**
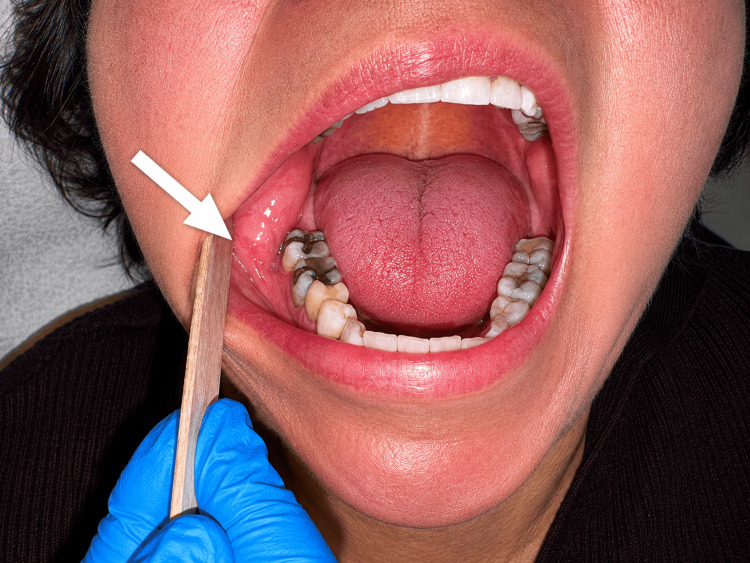
Intraoral correlation of the odontogenic cutaneous sinus tract Clinical intraoral photograph showing localized erythematous swelling of the buccal vestibule adjacent to the mandibular right first molar (#30). The tooth demonstrated extensive carious destruction and was identified as the source of a chronic periapical infection. The surrounding soft-tissue inflammation corresponded to the draining odontogenic cutaneous sinus tract observed extraorally. The white arrow highlights the area of vestibular swelling adjacent to the involved tooth.

Given the persistent nature of the mass and concern regarding potential neoplastic pathology, a panoramic radiograph was obtained. The radiology report described an enhancing soft tissue nodular density within the right mandibular region, measuring approximately 7 × 8 mm (Figure 3). Imaging demonstrated a sinus tract communicating directly with the right mandibular first molar. Associated findings included periapical radiolucency and focal cortical perforation of the right mandible. No suspicious submental or submandibular lymphadenopathy was identified. Additional cervical structures were reported as unremarkable. The radiologist concluded that the findings were most consistent with an OCST extending from the dental apex through the mandible to the skin surface.

**Figure 3:**
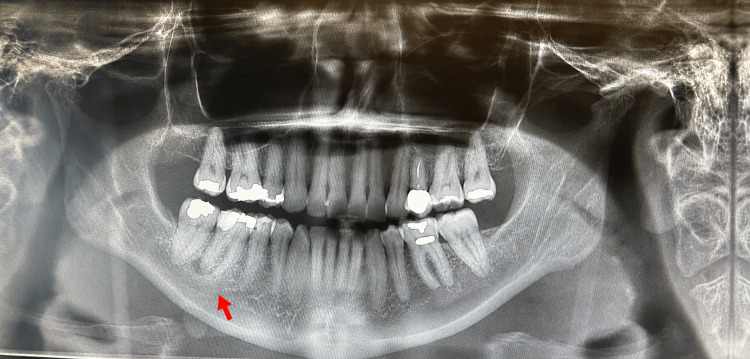
Radiographic correlation of the odontogenic cutaneous sinus tract Panoramic radiographic examination revealed extensive coronal destruction of the mandibular right first molar (#30) and a well-defined periapical radiolucency involving the root apices. The radiographic findings were consistent with a chronic odontogenic infection and correlated with the patient’s clinical presentation of an odontogenic cutaneous sinus tract. The red arrow indicates the area of periapical radiolucency adjacent to the involved tooth.

The differential diagnosis initially included cutaneous malignancy, chronic inflammatory nodule, epidermoid cyst, reactive lymphoid lesion, salivary gland pathology, and OCST. Radiographic demonstration of direct communication between the involved tooth and the cutaneous lesion established the diagnosis of an OCST of dental origin.

Following establishment of the diagnosis, the patient underwent extraction of the affected mandibular first molar with curettage of the associated periapical lesion. Tissue obtained during the procedure was submitted for histopathologic examination. Management was directed toward elimination of the chronic odontogenic infectious source responsible for formation of the cutaneous tract. At follow-up, the mandibular lesion demonstrated progressive clinical resolution without persistent drainage, recurrent infection, or interval enlargement. The favorable postoperative course further confirmed the odontogenic origin of the lesion.

## Discussion

OCSTs remain diagnostically challenging because they represent a dental disease process that often declares itself primarily through the skin. Although the underlying pathology is usually chronic pulpal necrosis with periapical infection, the clinical presentation may mimic a broad range of dermatologic, infectious, inflammatory, and neoplastic conditions. Large clinical series have shown that these lesions are frequently misdiagnosed because patients often present with cutaneous findings rather than dental complaints [1,2]. This diagnostic mismatch is particularly important because many patients initially seek evaluation from dermatologists, primary care physicians, otolaryngologists, general surgeons, plastic surgeons, or oncologic specialists before a dental source is considered [1,2].

The absence of significant dental pain is a major contributor to delayed recognition. Unlike acute odontogenic infections, OCSTs may decompress chronically through a sinus tract, reducing intramedullary pressure and producing minimal odontogenic symptoms despite persistent infection. As a result, the cutaneous lesion may appear clinically disproportionate to the patient’s dental history. Lee et al. noted that odontogenic cutaneous fistulas commonly appear as dimpling, nodules, or lesions with purulent discharge in the chin or jaw region, and that the lack of obvious dental symptoms can lead to misdiagnosis [2]. Similarly, Tian et al. described the case of a patient with periapical infection presenting primarily as a cutaneous lesion, further illustrating how odontogenic disease may masquerade as a nonodontogenic facial abnormality when dental symptoms are absent or subtle [3].

This case is notable because the lesion presented as a painless, persistent mandibular soft tissue mass rather than a classic draining sinus opening. This morphology broadened the clinical differential diagnosis and raised concern for non-dental pathology, including a possible neoplastic process. Odontogenic cutaneous fistulas have been reported to mimic malignancy, cysts, acneiform lesions, pyogenic granuloma, foreign body reactions, chronic osteomyelitis, actinomycosis, basal cell carcinoma, and squamous cell carcinoma. These diagnostic possibilities are clinically relevant because persistent nodular or ulcerative lesions of the face and neck are often approached first through a dermatologic or oncologic framework. In the present case, the nodular morphology, mandibular location, and persistence of the lesion appropriately prompted concern for malignancy; however, imaging ultimately demonstrated an odontogenic source.

Accurate identification of the odontogenic source was central to establishing the diagnosis in this case. Clinical examination demonstrated a chronic cutaneous lesion in conjunction with extensive carious destruction of the mandibular right first molar, while panoramic radiography revealed associated periapical pathology consistent with a chronic odontogenic infection. Correlation of the clinical and radiographic findings established the relationship between the dental infection and the extraoral lesion. Historically, gutta-percha tracing through a draining sinus tract has been used to confirm the odontogenic origin of cutaneous sinus tracts; however, this technique may be limited when lesions are nodular, intermittently draining, stenosed, or lack a clearly patent external opening. In such cases, careful clinical assessment combined with appropriate dental imaging remains essential for identifying the source of infection. Accurate diagnosis is particularly important because misidentification of OCSTs may lead to unnecessary dermatologic, medical, or surgical interventions before definitive dental treatment is provided [4]. The present case highlights how chronic odontogenic infection can mimic more concerning pathologies and emphasizes the importance of maintaining an odontogenic etiology in the differential diagnosis of persistent facial lesions.

The pathogenesis observed in this case is consistent with the established mechanism of OCST formation. Chronic pulpal necrosis leads to periapical inflammation and progressive osseous destruction. Once the infection perforates cortical bone, purulent material follows the path of least resistance through adjacent fascial planes and soft tissues until it establishes intraoral or extraoral drainage. The eventual cutaneous exit site depends on the involved tooth, root apex position, cortical plate thickness, muscle attachments, and fascial anatomy. Yadav et al. described a submental cutaneous sinus tract arising from a mandibular second molar, emphasizing that mandibular molar infections may drain to extraoral sites depending on the relationship between the root apices and surrounding anatomic barriers [5]. The present case similarly demonstrates how a mandibular molar infection may present externally as a mandibular or submandibular cutaneous lesion.

The broader literature also supports the importance of interdisciplinary awareness. Guevara-Gutiérrez et al. described the clinical and epidemiologic characteristics of 75 odontogenic cutaneous fistulas, highlighting the variable presentation and frequent delay in correct diagnosis [1]. Lee et al. similarly emphasized that extraoral and dental examinations are necessary for diagnosis and that cooperation between dermatologists and dentists is essential [2]. These observations are directly applicable to the present case, in which the patient’s lesion initially raised concern outside the dental framework. The case therefore reinforces the need for clinicians evaluating chronic facial or cervical lesions to include odontogenic infection in the differential diagnosis, particularly when the lesion is located near the mandibular border or when dental disease is evident clinically or radiographically.

Failure to recognize OCSTs can lead to prolonged antibiotic therapy, repeated incision and drainage, unnecessary biopsies, dermatologic excisions, scarring, cosmetic deformity, persistent infection, and increased healthcare utilization. Antibiotics alone are usually insufficient because they do not eliminate the necrotic pulp, periapical source, or infected tooth structure driving the sinus tract. Definitive management must therefore address the odontogenic origin. Depending on restorability and extent of disease, treatment may include nonsurgical root canal therapy, endodontic retreatment, apical surgery, extraction, or surgical management of the tract when indicated. Kelly and Murray emphasized that correct identification and treatment of the dental source are critical for resolution and avoiding unnecessary surgical or dermatologic intervention [4]. Latifa et al. similarly noted that management should focus on eliminating the underlying odontogenic infection, although persistent or recurrent lesions may require surgical intervention to achieve complete resolution and optimal cosmetic healing [6].

The prognosis of OCSTs is generally favorable once the odontogenic source is eliminated. Numerous reports have demonstrated spontaneous resolution of the cutaneous lesion following definitive treatment of the affected tooth, often without the need for extensive dermatologic intervention. Nevertheless, delayed diagnosis may result in residual fibrosis, cutaneous scarring, or cosmetic deformity despite eradication of the infection. Therefore, prompt recognition and treatment are important not only for infection control but also for minimizing long-term esthetic consequences and reducing unnecessary healthcare utilization [7].

This case contributes to the existing literature by emphasizing a neoplasm-mimicking presentation of OCST in which a chronic odontogenic infection presented as a persistent mandibular soft tissue mass rather than a classic draining sinus. The case also demonstrates the diagnostic utility of radiographic evaluation when clinical examination alone is insufficient and when the differential diagnosis includes malignancy, lymphadenopathy, or other nonodontogenic pathology. For clinicians, the key lesson is that chronic facial nodules, draining lesions, or unexplained mandibular cutaneous masses should prompt careful dental examination and radiographic correlation before invasive dermatologic or oncologic procedures are pursued. Early recognition of the odontogenic source allows definitive treatment, prevents unnecessary interventions, and may reduce morbidity associated with delayed diagnosis.

## Conclusions

OCSTs should remain an important consideration in the differential diagnosis of persistent facial lesions, draining sinus tracts, and chronic mandibular masses, particularly when conventional treatments fail to achieve resolution. This case highlights the potential for chronic odontogenic infections to mimic neoplastic processes and underscores the importance of correlating clinical findings with dental radiographic evaluation. Early recognition of the underlying dental source can prevent diagnostic delays, avoid unnecessary procedures, and facilitate timely definitive treatment. Greater awareness of this uncommon presentation among both dental and medical practitioners may improve diagnostic accuracy and patient outcomes.
